# Prognostic value of the Geriatric Nutritional Risk Index in patients undergoing cardiac surgery: a systematic review and meta-analysis

**DOI:** 10.3389/fnut.2025.1628671

**Published:** 2025-07-25

**Authors:** Ping Luo, Kai Shi, Yu Luo, Hai-Bo Ren

**Affiliations:** Wuhan Asia Heart Hospital, Wuhan, China

**Keywords:** cardiac surgery, Geriatric Nutritional Risk Index, meta-analysis, mortality, postoperative complications

## Abstract

**Background:**

The Geriatric Nutritional Risk Index (GNRI) is a key indicator of nutritional status in elderly individuals. Poor nutritional status has been linked to unfavorable surgical outcomes, but its prognostic value in cardiac procedures remains uncertain. This meta-analysis investigates the relationship between the GNRI and prognosis in cardiac surgery patients.

**Methods:**

A comprehensive literature search was performed across the PubMed, Embase, and Web of Science databases. Studies were included if they evaluated preoperative GNRI and reported short-term mortality, long-term mortality, or major postoperative complications, such as acute kidney injury (AKI), wound complications, and infections. Risk ratios (RRs) and 95% confidence intervals (CIs) were calculated to compare outcomes between patients with low and normal GNRI. Heterogeneity was assessed using the *I*^2^ statistic, and a random-effects model was used to synthesize and analyze the results from the included studies.

**Results:**

The pooled results of 16 cohort studies involving 7,593 patients showed that a low preoperative GNRI was significantly associated with an increased risk of short-term mortality (RR: 3.19, 95% CI: 1.68–6.07, *p* < 0.001; *I*^2^ = 39%) and long-term mortality (RR: 2.32, 95% CI: 1.63–3.30, *p* < 0.001; *I*^2^ = 77%). Low GNRI was correlated with a higher risk of AKI (RR: 1.77, 95% CI: 1.11–2.81, *p* = 0.02; *I*^2^ = 74%) and overall infection (RR: 3.35, 95% CI: 2.01–5.57, *p* < 0.001; *I*^2^ = 29%), while no significant association was observed for wound complications, although this outcome was based on only four studies. Meta-regression identified mean age as a significant contributor to heterogeneity in long-term mortality (*p* = 0.04), while sample size explained part of the heterogeneity in short-term mortality (adjusted *R*^2^ = 23.5%).

**Conclusion:**

A low preoperative GNRI is correlated with an increased risk of mortality and postoperative complications in cardiac surgery patients. Preoperative nutritional assessment using GNRI may identify high-risk patients.

**Systematic review registration:**

PROSPERO ID CRD42025637322.

## Introduction

With the growing prevalence of cardiovascular diseases, like coronary artery and valvular heart disease, cardiac surgery is becoming more common globally, especially among aging populations (1, 2). Although advancements in surgical techniques and perioperative care have enhanced survival rates, cardiac surgery still carries substantial risks (3). Postoperative complications, including acute kidney injury (AKI), infections, and wound complications, as well as short- and long-term mortality, pose substantial challenges to improving patient outcomes (4, 5). These adverse events not only increase morbidity and healthcare costs but also negatively impact the functional recovery and long-term quality of life of the patients (6). Identifying novel, reliable predictors of adverse postoperative outcomes is essential for risk stratification and the optimization of perioperative management (6).

Malnutrition has emerged as a critical determinant of postoperative outcomes in cardiac surgery (7). Poor nutritional status has been associated with compromised immune function, delayed wound healing, muscle wasting, and increased susceptibility to infections, all of which can contribute to poor recovery and increased mortality (8, 9). Malnutrition is particularly prevalent in older patients undergoing cardiac surgery due to age-related physiological changes, chronic diseases, and reduced dietary intake (10).

To assess the nutritional status of patients, various biomarkers and scoring systems have been established, with the Geriatric Nutritional Risk Index (GNRI) gaining recognition as a practical and prognostic tool (8). The GNRI is a straightforward and objective index calculated from serum albumin levels and body weight in relation to ideal weight (11). It was initially developed to assess nutritional risk in elderly patients and has since been applied to various clinical settings, including cardiac surgery (12). Compared to other nutritional assessment tools, the GNRI offers several advantages: it is easy to calculate, does not require complex measurements such as muscle mass evaluation, and has been validated as a predictor of adverse outcomes in hospitalized patients (13, 14). Importantly, the GNRI can be readily incorporated into routine preoperative assessments, allowing for the early identification of high-risk patients and the implementation of targeted nutritional interventions (15).

Recent studies have investigated the association between the GNRI and postoperative outcomes after cardiac surgery. However, the results of these studies are not always consistent, leading to uncertainty regarding the clinical utility of the GNRI in this context (16–31). Variations in study designs, sample sizes, GNRI cutoff values, and outcome definitions may have contributed to these discrepancies. Given the potential impact of malnutrition on surgical outcomes and the need for reliable prognostic tools, a comprehensive synthesis and analysis of the available evidence is warranted. Accordingly, this meta-analysis was conducted to examine the relationship between the GNRI and postoperative outcomes in patients undergoing cardiac surgery.

## Methods

This meta-analysis followed the Preferred Reporting Items for Systematic Reviews and Meta-Analyses (PRISMA) 2020 guidelines (32, 33) and the Cochrane Handbook for Systematic Reviews and Meta-Analyses (34) for protocol design, data extraction, statistical analysis, and results reporting. The study protocol was also registered in the International Prospective Register of Systematic Reviews database, also known as PROSPERO, under ID CRD42025637322.

### Literature search

Relevant studies for this meta-analysis were identified through a comprehensive search across the PubMed, Embase, and Web of Science databases using a broad range of search terms, including: (“geriatric nutritional risk index” OR “GNRI”) AND (“cardiac surgery” OR “heart surgery” OR “coronary artery bypass” OR “CABG” OR “cardiopulmonary bypass” OR “valve surgery” OR “tricuspid valve replacement” OR “mitral valve replacement” OR “aortic valve replacement” OR “cardiopulmonary bypass”). The search was limited to human studies and full-length articles published in English in peer-reviewed journals. Additionally, references from relevant original and review articles were manually screened for identifying further eligible studies. The search spanned from database inception to December 9, 2024.

### Inclusion and exclusion criteria

The eligibility criteria for studies were established based on the PICOS framework:

P (patients): Adult patients undergoing cardiac surgery, such as coronary artery bypass grafting (CABG) and valvular surgeries.

I (exposure): Patients identified as malnourished as indicated by a low GNRI at baseline, with cutoff values consistent with those used in the original studies.

C (comparison): Patients with a normal GNRI at baseline.

O (outcome): Incidence of short-term mortality, defined as all-cause deaths occurring during hospitalization or within 3 months; the incidence of postoperative complications, such as AKI, wound-related complications, and overall infection, as defined by the criteria of the included studies; and long-term mortality, defined as all-cause deaths with a follow-up duration of at least 1 year.

S (study design): Longitudinal observational studies, including cohort studies, nested case–control studies, and post-hoc analyses of clinical trials.

Studies were excluded if they were reviews, editorials, meta-analyses, or preclinical research, or if they did not focus specifically on patients undergoing cardiac surgery, were lacking in GNRI exposure assessment, or did not report any of the outcomes of interest. Studies involving patients undergoing cardiac transplantation were also excluded, as this patient population has a distinct disease course and prognosis compared to those undergoing conventional cardiac surgeries, such as CABG or valvular procedures. In cases of population overlap, the study with the largest sample size was selected for inclusion in the meta-analysis.

### Study quality assessment and data extraction

Two authors independently conducted the literature search, study selection, quality assessment, and data extraction, resolving discrepancies through discussion with the corresponding author. Study quality was evaluated using the Newcastle–Ottawa Scale (NOS) (35), which assesses selection, confounding control, and outcome measurement, with scores ranging from 1 to 9, where 9 represents the highest quality studies. Studies with NOS scores of 7 or above are considered of high quality. The data extracted for analysis included the following study characteristics: author, year, country, and design; type of surgery, participant details (number of included patients, mean age, and sex); timing of GNRI assessment; methods used to determine the cutoff for the GNRI; the cutoff value of the GNRI indicative of malnutrition; the number of patients classified as malnourished based on a low GNRI; follow-up durations; reported outcomes; and the analytical model used (univariate or multivariate) to estimate the relationship between the GNRI and outcomes following cardiac surgery.

### Statistical analyses

The primary outcome of this study was to determine the association between the GNRI and short-term (within 90 days) and long-term (beyond 1 year) mortality in patients after cardiac surgery. The secondary outcome was to evaluate the association between the GNRI and the incidence of AKI, wound complications, and overall infection during hospitalization. The results were expressed as risk ratios (RRs) with 95% confidence intervals (CIs), comparing the outcome incidence between the patients with a low GNRI and those with a normal GNRI. RRs and their standard errors were calculated from 95% CIs or *p*-values and log-transformed to stabilize variance and normalize distribution (34). To assess heterogeneity, we used the Cochrane Q test and *I*^2^ statistics (36), with *I*^2^ < 25, 25–75%, and >70% indicating mild, moderate, and substantial heterogeneity among the included studies, respectively. A random-effects model was used to synthesize and analyze the results while accounting for variability across studies (34). Sensitivity analysis was conducted by sequentially excluding individual studies to assess the robustness of the findings. For the primary outcome, subgroup analyses were performed to explore the effects of various factors on the results, such as type of surgery (CABG, valvular surgery, or overall type of cardiac surgery), cutoffs of the GNRI, follow-up durations, analytic models, and NOS scores. Subgroups were defined using the median values of continuous variables as cutoff points. Publication bias was assessed through funnel plots, visual asymmetry inspection, and Egger’s regression test (37). In addition, univariate meta-regression analyses were performed to evaluate if the association between the GNRI and short-term or long-term mortality risk of patients after cardiac surgery could be significantly affected by continuous variables in the included studies, such as sample size, mean age, proportion of men, cutoff values of the GNRI, or NOS scores (34). A *p-*value < 0.05 indicated statistical significance. The statistical analyses were conducted using RevMan (version 5.1; Cochrane Collaboration, Oxford, UK) and Stata software (version 12.0; Stata Corporation, College Station, TX, USA).

## Results

### Study identification

**Figure 1:**
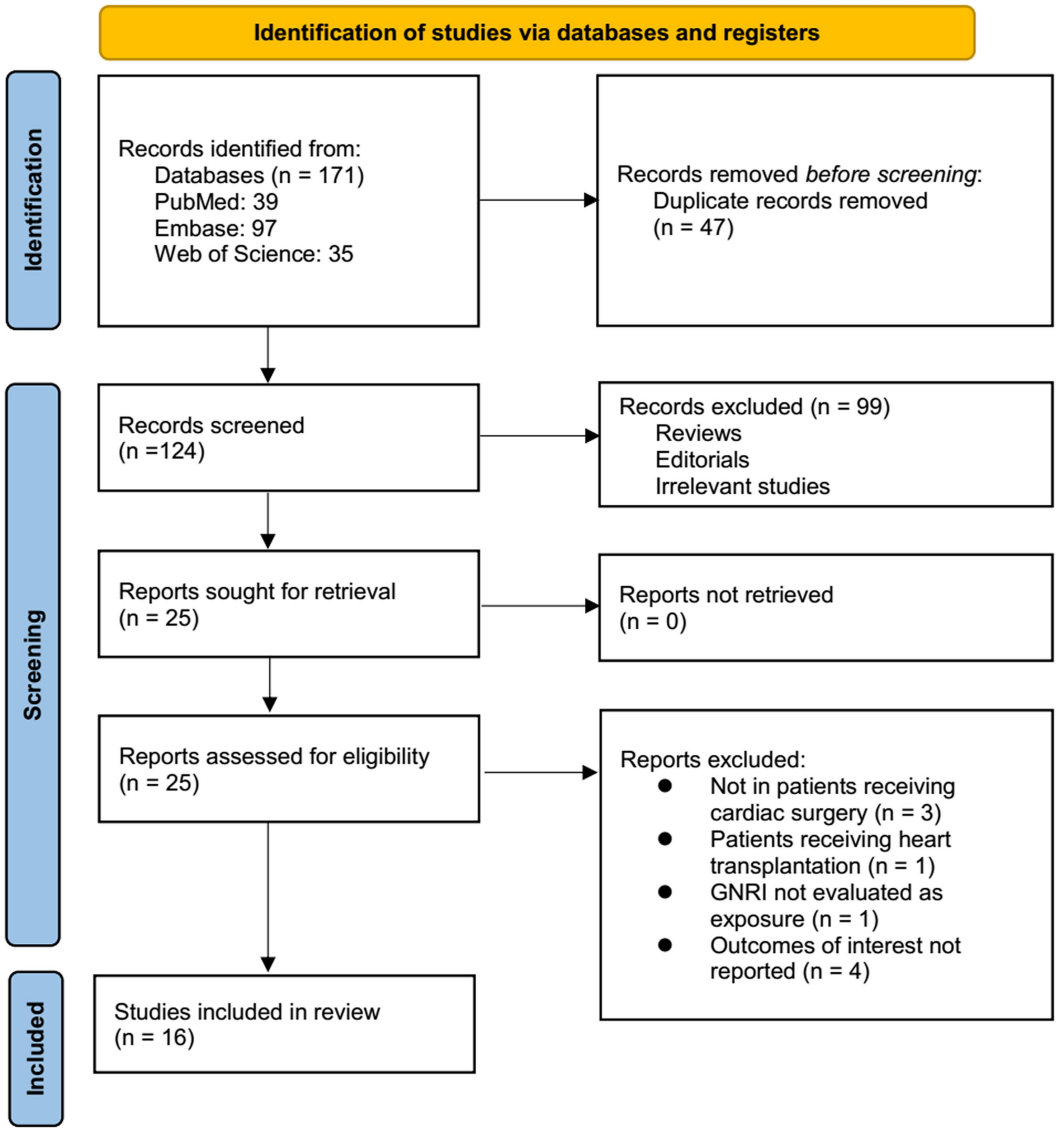
Flowchart of the database searches and study inclusion.

Figure 1 outlines the study selection process. Initially, 171 records were identified across the three databases, with 47 duplicates removed. After title and abstract screening, 99 articles were further excluded for not meeting the meta-analysis criteria. The full texts of the remaining 25 studies were independently reviewed by two authors, leading to the further exclusion of 9 for the reasons detailed in Figure 1. Ultimately, 16 studies were included in the quantitative analysis (16–31).

### Overview of the study characteristics

Table 1 presents a summary of the characteristics of the studies included in the meta-analysis. Overall, one prospective cohort study (19) and 15 retrospective cohort studies (16–18, 20–31) were included in the meta-analysis. These studies were published from 2019 to 2024, and were conducted in Japan, Hungary, Turkey, Korea, China, Italy, and the United States. A total of 7,593 patients undergoing cardiac surgeries, such as CABG and/or valvular surgeries, were included. The mean ages of the patients were 59.6 to 82.1 years old, and the proportions of men were 44.7–82.2%. All the patients were assessed for GNRI before cardiac surgery. The cutoff values for a low GNRI were based on previously defined cutoffs in 13 studies (16, 19–23, 25–31), as well as the receiver operating characteristic curve analysis-derived cutoffs in three studies (17, 18, 24). The cutoff values for defining a low GNRI varied from 91.0 to 102.5. Follow-up periods ranged from in the hospitalization period to 4.5 years after. The outcome of short-term mortality was reported in 10 studies (16, 17, 22, 23, 25, 27–31). The incidences of postoperative AKI, wound complications, and overall infection were reported in seven (16, 20, 24, 27–29, 31), four (16, 22, 27, 29), and five studies (16, 22, 25, 27, 31), respectively. The outcome of long-term mortality was reported in nine studies (17–19, 21–23, 26, 29, 30). NOS scores ranged from six to nine, indicating moderate to high methodological and reporting quality (Table 2).

**Table 1:** Characteristics of the included studies.

Study	Country	Design	Surgery	No. of patients	Mean age (years)	Men (%)	Timing of GNRI assessment	Methods for determining the cutoff of GNRI	Cutoff value of GNRI for malnutrition	No. of patients with malnutrition	Follow-up duration	Outcomes reported	Analytic models
Unosawa et al. (2019) (16)	Japan	RC	Elective cardiac surgery	287	68.9	73.9	Preoperative	Previous study defined cutoff	<91	51	During hospitalization	Postoperative AKI, overall infection, wound complications, and mortality	Multivariate analysis for postoperative death, univariate analysis for other outcomes
Tóth et al. (2021) (19)	Hungary	PC	Elective cardiac surgery	69	65.3	63.8	Preoperative	Previous study defined cutoff	<91	NR	4.5 years	Long-term mortality	Multivariate analysis
Tasbulak et al. (2021) (18)	Turkey	RC	Isolated CABG	586	59.6	77.8	At admission	ROC analysis derived	NR	NR	3.2 years	Long-term mortality	Univariate analysis
Gürbak et al. (2021) (17)	Turkey	RC	SAVR	150	70	52	Preoperative	ROC analysis derived	<102.5	51	4.2 years	Inhospital and long-term mortality	Multivariate analysis
Usta and Engin (2022) (24)	Turkey	RC	Cardiac surgery under CPB	439	69.6	77.7	Preoperative	ROC analysis derived	<93.6	NR	During hospitalization	Postoperative AKI	Multivariate analysis
Naganuma et al. (2022) (22)	Japan	RC	SAVR	219	74	44.7	Preoperative	Previous study defined cutoff	<98	58	2.7 years	Postoperative overall infection, wound complications, inhospital mortality, and long-term mortality	Multivariate analysis for long-term mortality, univariate analysis for other outcomes
Aykut and Salman (2022) (20)	Turkey	RC	On-pump CABG	455	61.3	82.2	Preoperative	Previous study defined cutoff	<91	11	During hospitalization	Postoperative AKI	Multivariate analysis
Tóth et al. (2022) (23)	Hungary	PC	Elective cardiac surgery	252	64.2	66.3	Preoperative	Previous study defined cutoff	<91	15	1.7 years	Short-term (30-day) and long-term mortality	Multivariate analysis
Cho et al. (2022) (21)	Korea	RC	Valvular heart surgery	1927	63	51.1	Preoperative	Previous study defined cutoff	<98	590	1 year	Long-term mortality	Univariate analysis
Çoban and Uçak (2023) (25)	Turkey	RC	Open heart surgery	22	82.1	59.1	Preoperative	Previous study defined cutoff	<98	6	During hospitalization	Postoperative overall infection and mortality	Univariate analysis
Wu et al. (2023) (27)	China	RC	Elective cardiac surgery	292	59.8	64	Within 48 h after admission	Previous study defined cutoff	<98	136	90 days	Postoperative AKI, overall infection, wound complications, and short-term mortality	Univariate analysis
Liu et al. (2023) (26)	China	RC	Cardiac surgery	401	69	65.3	Preoperative	Previous study defined cutoff	<98	60	3.3 years	Long-term mortality	Univariate analysis
Takagi et al. (2024) (30)	Japan	RC	Off-pump CABG	632	69	79.5	Preoperative	Previous study defined cutoff	<98	155	5 years	Short-term (30-day) and long-term mortality	Univariate analysis
Pavone et al. (2024) (29)	Italy	RC	Isolated heart valve surgery	448	77.6	54.9	Preoperative	Previous study defined cutoff	<92	102	2.7 years	Postoperative AKI, wound complications, and short-term (30-day) and long-term mortality	Univariate analysis
Bao et al. (2024) (28)	USA	RC	CABG	1,007	73.5	73.1	Preoperative	Previous study defined cutoff	<98	100	During hospitalization	Postoperative AKI and short-term mortality	Univariate analysis for mortality, multivariate analysis for AKI
Zhao et al. (2024) (31)	China	RC	Cardiac surgery	407	66.2	54.8	Preoperative	Previous study defined cutoff	<98	278	During hospitalization	Postoperative AKI, overall infection, and mortality	Univariate analysis

AKI, acute kidney injury; CABG, coronary artery bypass grafting; CPB, cardiopulmonary bypass; GNRI, Geriatric Nutritional Risk Index; NR, not reported; PC: prospective cohort; RC: retrospective cohort; ROC: receiver operating characteristic; SAVR, surgical aortic valve replacement.

**Table 2:** Study quality evaluation via the Newcastle–Ottawa Scale.

Study	Representativeness of the exposed cohort	Selection of the non-exposed cohort	Ascertainment of exposure	Outcome not present at baseline	Control for age and sex	Control for other confounding factors	Assessment of outcome	Enough long follow-up duration	Adequacy of follow-up of cohorts	Total
Unosawa et al. (2019) (16)	0	1	1	1	0	0	1	1	1	6
Tóth et al. (2021) (19)	1	1	1	1	1	1	1	1	1	9
Tasbulak et al. (2021) (18)	1	1	1	1	0	0	1	1	1	7
Gürbak et al. (2021) (17)	1	1	1	1	1	1	1	1	1	9
Usta and Engin (2022) (24)	1	1	1	1	1	1	1	1	1	9
Naganuma et al. (2022) (22)	0	1	1	1	1	1	1	1	1	8
Aykut and Salman (2022) (20)	0	1	1	1	1	1	1	1	1	8
Tóth et al. (2022) (23)	1	1	1	1	1	1	1	1	1	9
Cho et al. (2022) (21)	0	1	1	1	0	0	1	1	1	6
Çoban and Uçak (2023) (25)	0	1	1	1	0	0	1	1	1	6
Wu et al. (2023) (27)	0	1	1	1	0	0	1	1	1	6
Liu et al. (2023) (26)	0	1	1	1	0	0	1	1	1	6
Takagi et al. (2024) (30)	0	1	1	1	0	0	1	1	1	6
Pavone et al. (2024) (29)	1	1	1	1	0	0	1	1	1	7
Bao et al. (2024) (28)	0	1	1	1	1	1	1	1	1	8
Zhao et al. (2024) (31)	0	1	1	1	0	0	1	1	1	6

### GNRI and short-term mortality after cardiac surgery

**Figure 2:**
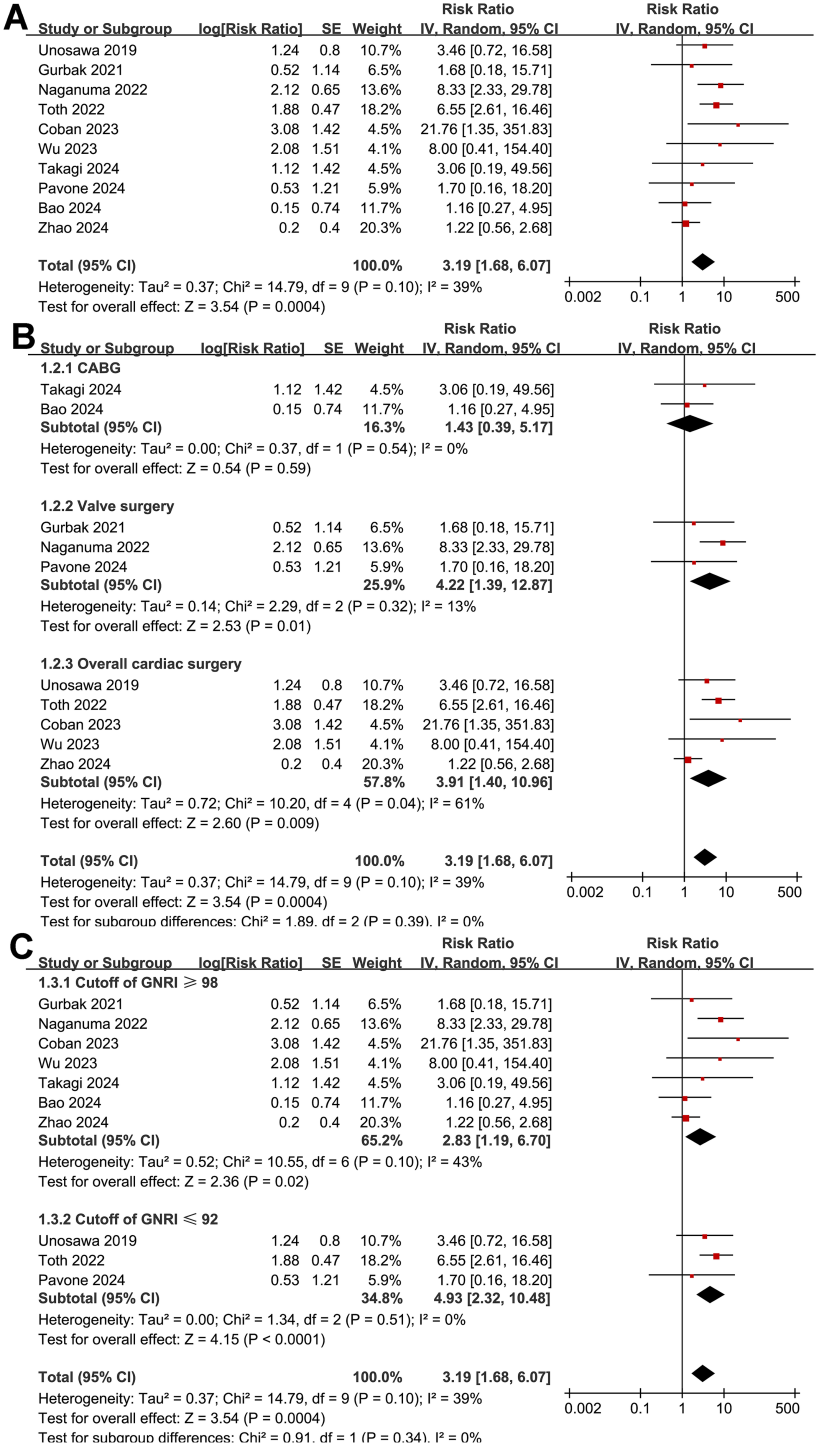
Forest plots for the meta-analysis of the association between a low GNRI and the risk of short-term mortality in patients after cardiac surgery. **(A)** Overall meta-analysis; **(B)** subgroup analysis according to the surgery type; **(C)** subgroup analysis according to the cutoffs of the GNRI. GNRI, Geriatric Nutritional Risk Index.

**Figure 3:**
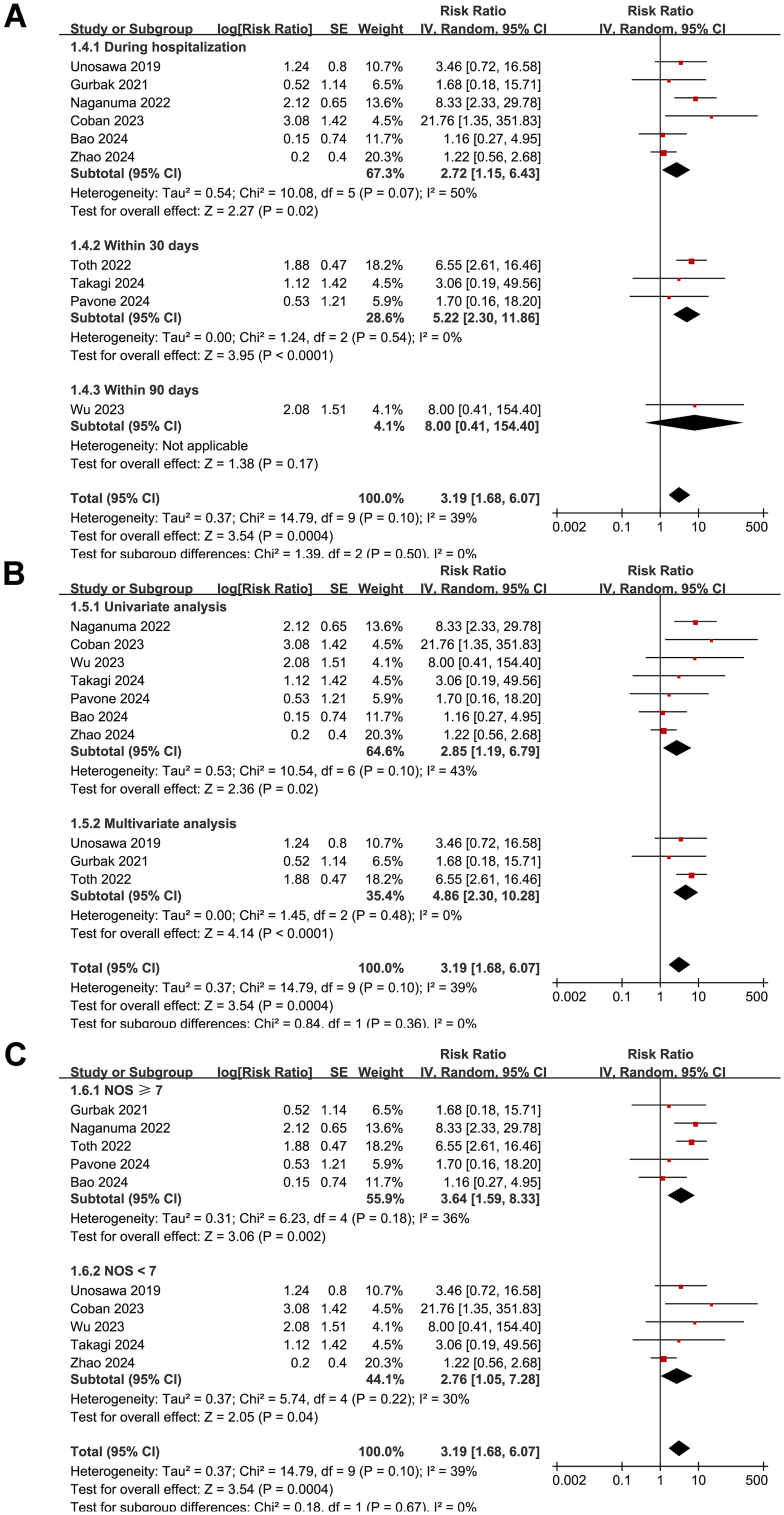
Forest plots for the subgroup analyses of the association between a low GNRI and the risk of short-term mortality in patients after cardiac surgery. **(A)** Subgroup analysis according to the follow-up durations; **(B)** subgroup analysis according to the analytic models; **(C)** subgroup analysis according to the NOS scores. NOS, Newcastle–Ottawa Scale.

Overall, ten studies (16, 17, 22, 23, 25, 27–31) reported the association between a low preoperative GNRI and the risk of short-term mortality after cardiac surgery. The pooled results indicated that a low GNRI, reflecting malnutrition, was linked to a higher risk of postoperative mortality within 90 days of cardiac surgery (RR: 3.19, 95% CI: 1.68–6.07, *p* < 0.001; *I*^2^ = 39%; Figure 2A). Sensitivity analysis, excluding one study at a time, showed no significant impact on the results (RR: 2.72–4.40, *p* all < 0.05). Subsequent subgroup analyses suggested that the association between a low GNRI and short-term mortality after cardiac surgery was not significantly modified by the type of cardiac surgery (*p* for subgroup difference = 0.39; Figure 2B), cutoffs of the GNRI (*p* for subgroup difference = 0.34; Figure 2C), follow-up durations (*p* for subgroup difference = 0.50; Figure 3A), analytic models (*p* for subgroup difference = 0.36; Figure 3B), or study quality scores (*p* for subgroup difference = 0.67; Figure 3C). Consistently, univariate meta-regression analysis also did not suggest that sample size, mean age, proportion of men, cutoff values of the GNRI, or NOS scores of the included studies significantly affected the association between the GNRI and short-term mortality of patients after cardiac surgery (*p* all >0.05; Table 3). However, among these factors, differences in the sample size may explain the majority of the heterogeneity, with an adjusted *R*^2^ = 23.5% (*p* = 0.095).

**Table 3:** Results of the univariate meta-regression analyses.

Variables	RR for the association between GNRI and short-term mortality				RR for the association between GNRI and long-term mortality			
	Coefficient	95% CI	*p*-value	Adjusted *R*^2^	Coefficient	95% CI	*p*-value	Adjusted *R*^2^
Sample size	−0.0022	−0.0049 to 0.0005	0.095	23.5%	0.00022	−0.00082 to 0.00126	0.63	0%
Mean age (years)	0.017	−0.135 to 0.170	0.80	0%	−0.090	−0.17 to −0.01	0.04	63.0%
Men (%)	−0.010	−0.088 to 0.067	0.77	0%	−0.0080	−0.0633 to 0.0743	0.74	0%
Cutoff of GNRI	−0.072	−0.279 to 0.136	0.45	9.6%	0.021	−0.141 to 0.184	0.76	0%
NOS	0.16	−0.43 to 0.76	0.54	10.1%	0.24	−0.19 to 0.68	0.21	6.9%

RR, relative risk; CI, confidence interval; GNRI, Geriatric Nutritional Risk Index; NOS, Newcastle–Ottawa Scale.

### GNRI and postoperative complications

**Figure 4:**
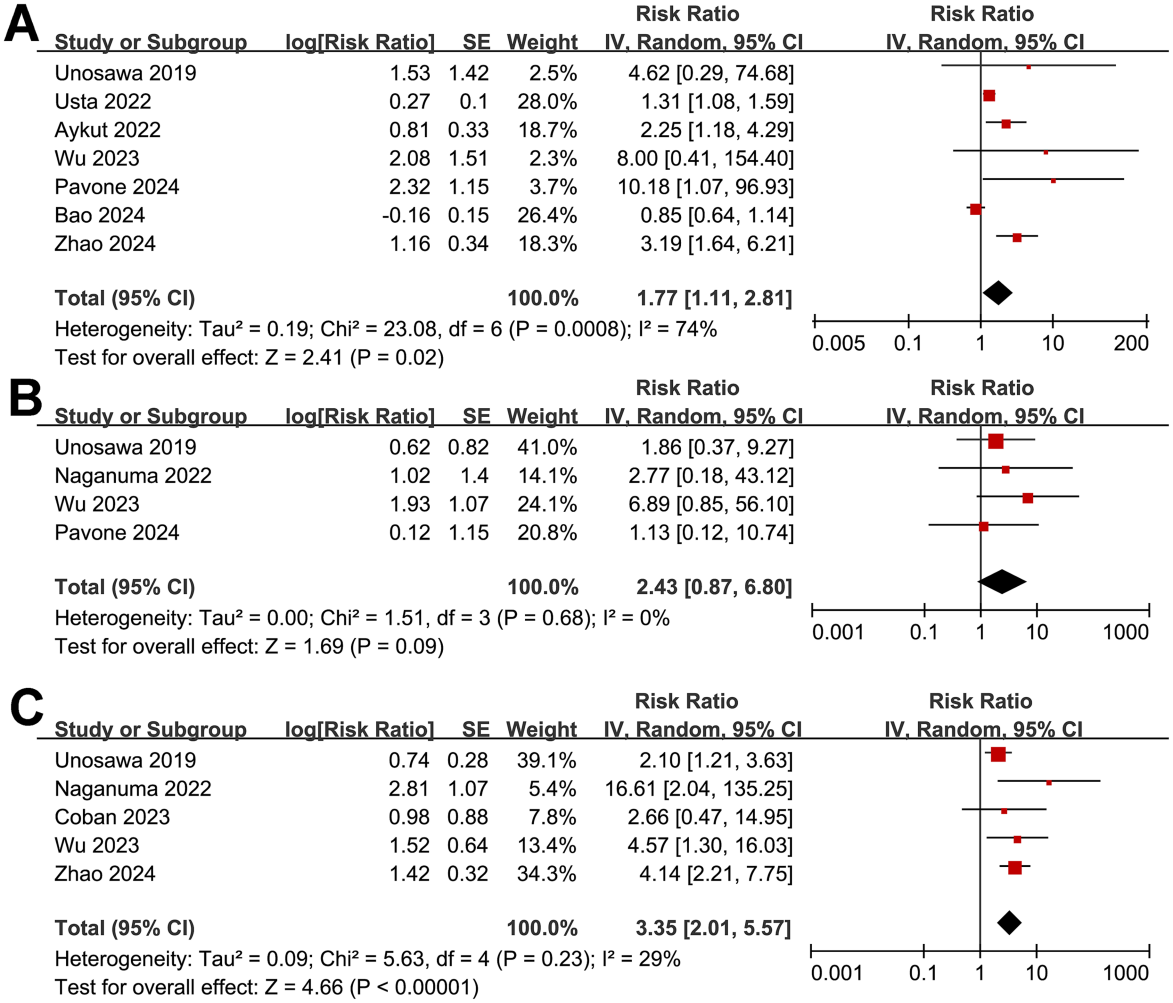
Forest plots for the meta-analysis of the association between a low GNRI and the risk of postoperative complications. **(A)** Forest plots for the outcome of postoperative AKI; **(B)** forest plots for the outcome of overall wound complications; **(C)** forest plots for the outcome of overall infection after cardiac surgery. AKI, acute kidney injury; GNRI, Geriatric Nutritional Risk Index.

Meta-analysis with seven studies (16, 20, 24, 27–29, 31) suggested that a low GNRI before surgery was associated with a higher risk of AKI after cardiac surgery (RR: 1.77, 95% CI: 1.11–2.81, *p* = 0.02; *I*^2^ = 74%; Figure 4A), while the pooled results of four studies (16, 22, 27, 29) did not show a significant association between a low GNRI and an increased incidence of wound complications (RR: 2.43, 95% CI: 0.87–6.80, *p* = 0.09; *I*^2^ = 0%; Figure 4B). Further meta-analysis with five studies (16, 22, 25, 27, 31) suggested that malnutrition as indicated by a low GNRI was associated with an increased risk of overall infection after cardiac surgery (RR: 3.35, 95% CI: 2.01–5.57, *p* < 0.001; *I*^2^ = 29%; Figure 4C).

### GNRI and long-term mortality after cardiac surgery

**Figure 5:**
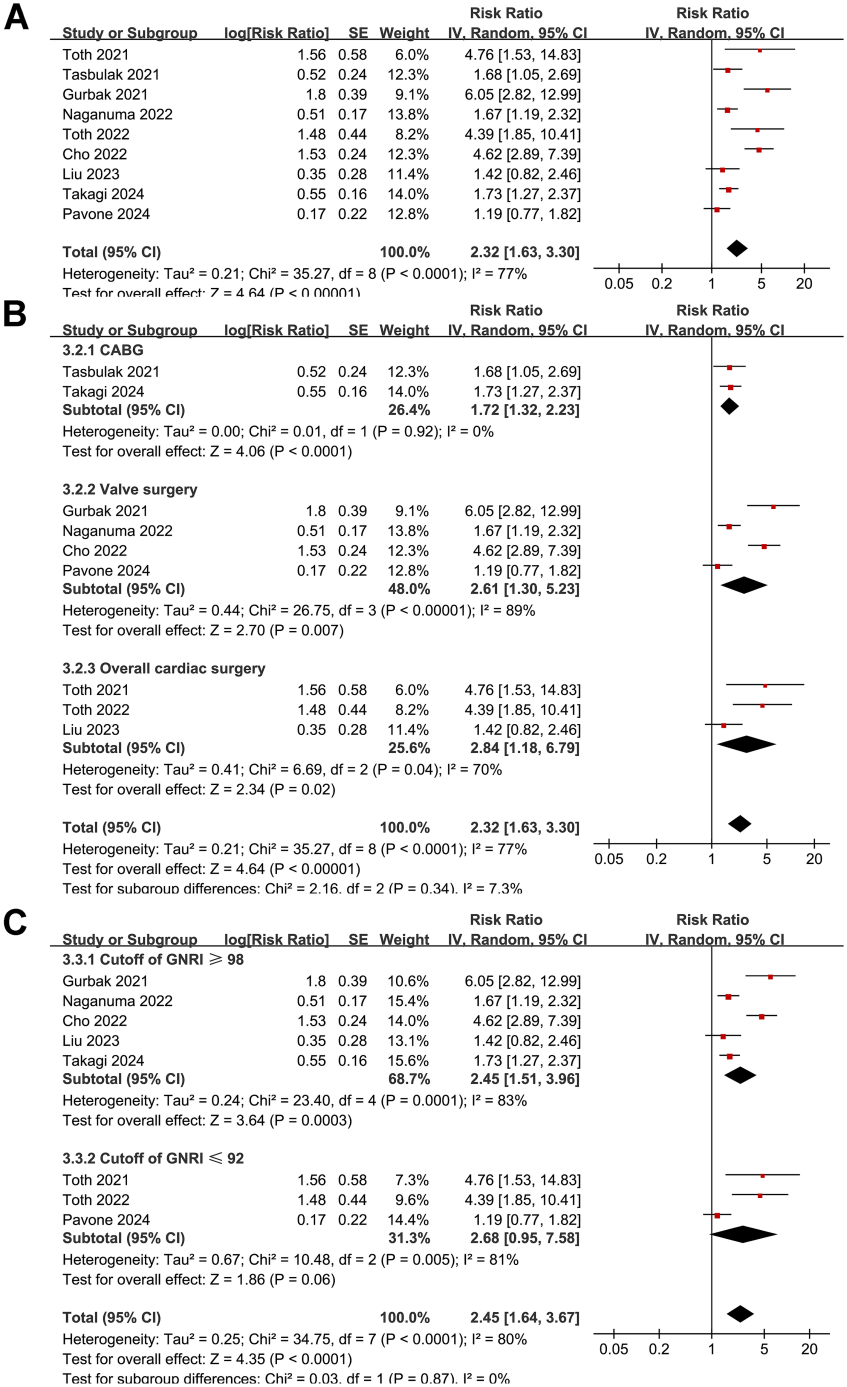
Forest plots for the meta-analysis of the association between a low GNRI and the risk of long-term mortality in patients after cardiac surgery. **(A)** Overall meta-analysis; **(B)** subgroup analysis according to the surgery type; **(C)** subgroup analysis according to the cutoffs of GNRI. GNRI, Geriatric Nutritional Risk Index.

**Figure 6:**
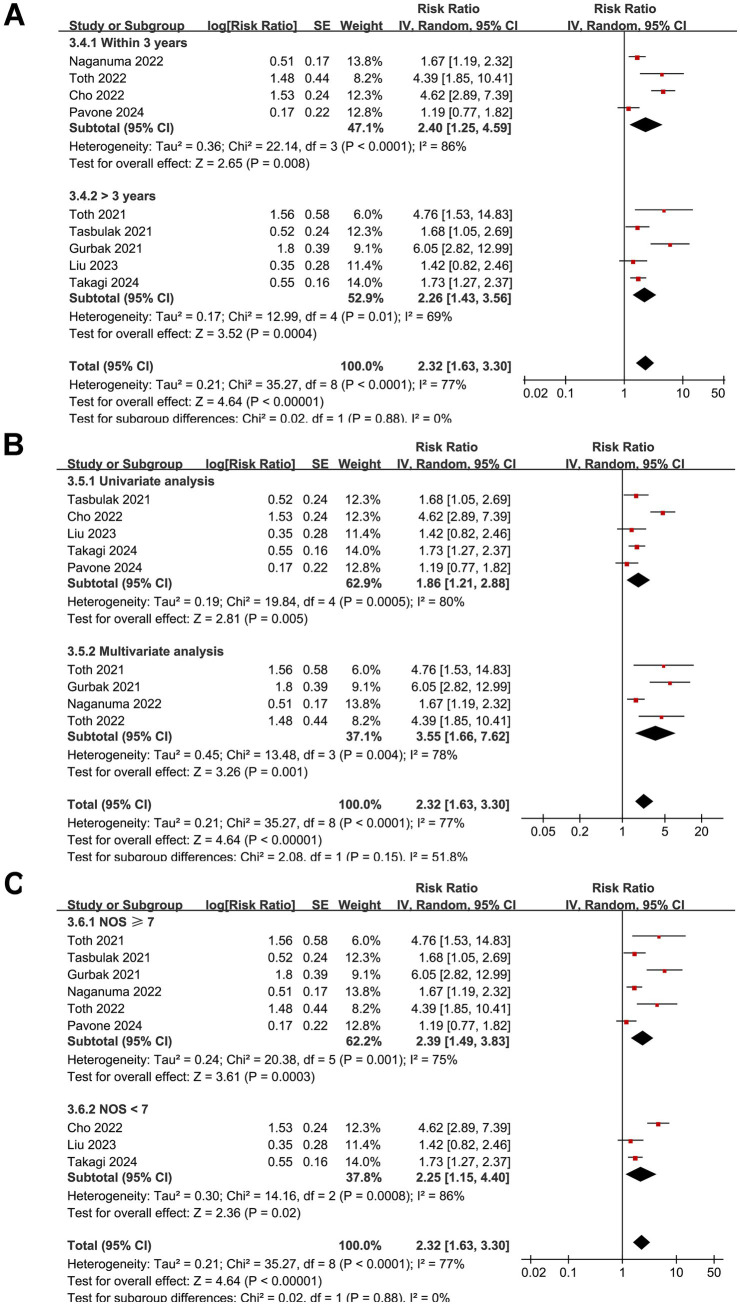
Forest plots for the subgroup analyses of the association between a low GNRI and the risk of long-term mortality in patients after cardiac surgery. **(A)** Subgroup analysis according to the follow-up durations; **(B)** subgroup analysis according to the analytic models; **(C)** subgroup analysis according to the NOS scores.

The pooled results of nine studies (17–19, 21–23, 26, 29, 30) showed that a low GNRI before surgery was associated with an increased risk of long-term mortality in patients after cardiac surgery (RR: 2.32, 95% CI: 1.63–3.30, *p* < 0.001; *I*^2^ = 77%; Figure 5A). Omitting individual studies in the sensitivity analysis did not significantly alter the results (RR: 2.03–2.55, *p* all < 0.05). Further subgroup analyses according to the type of surgery (*p* for subgroup difference = 0.34; Figure 5B), cutoffs of the GNRI (*p* for subgroup difference = 0.87; Figure 5C), follow-up durations (*p* for subgroup difference = 0.88; Figure 6A), analytic models (*p* for subgroup difference = 0.15; Figure 6B), or NOS scores (p for subgroup difference = 0.88; Figure 6C) did not significantly affect the association between the GNRI and long-term mortality. Finally, univariate meta-regression analysis indicated that the mean ages of the included patients were negatively correlated with the RR for the association between the GNRI and long-term mortality (coefficient = −0.090, *p* = 0.04; Table 3), which could explain the substantial between-study heterogeneity (adjusted *R*^2^ = 63.0%). Meanwhile, other factors, such as sample size, proportion of men, cutoff of the GNRI, or NOS score, did not significantly affect the association (*p* all > 0.05; Table 3).

### Publication bias

**Figure 7:**
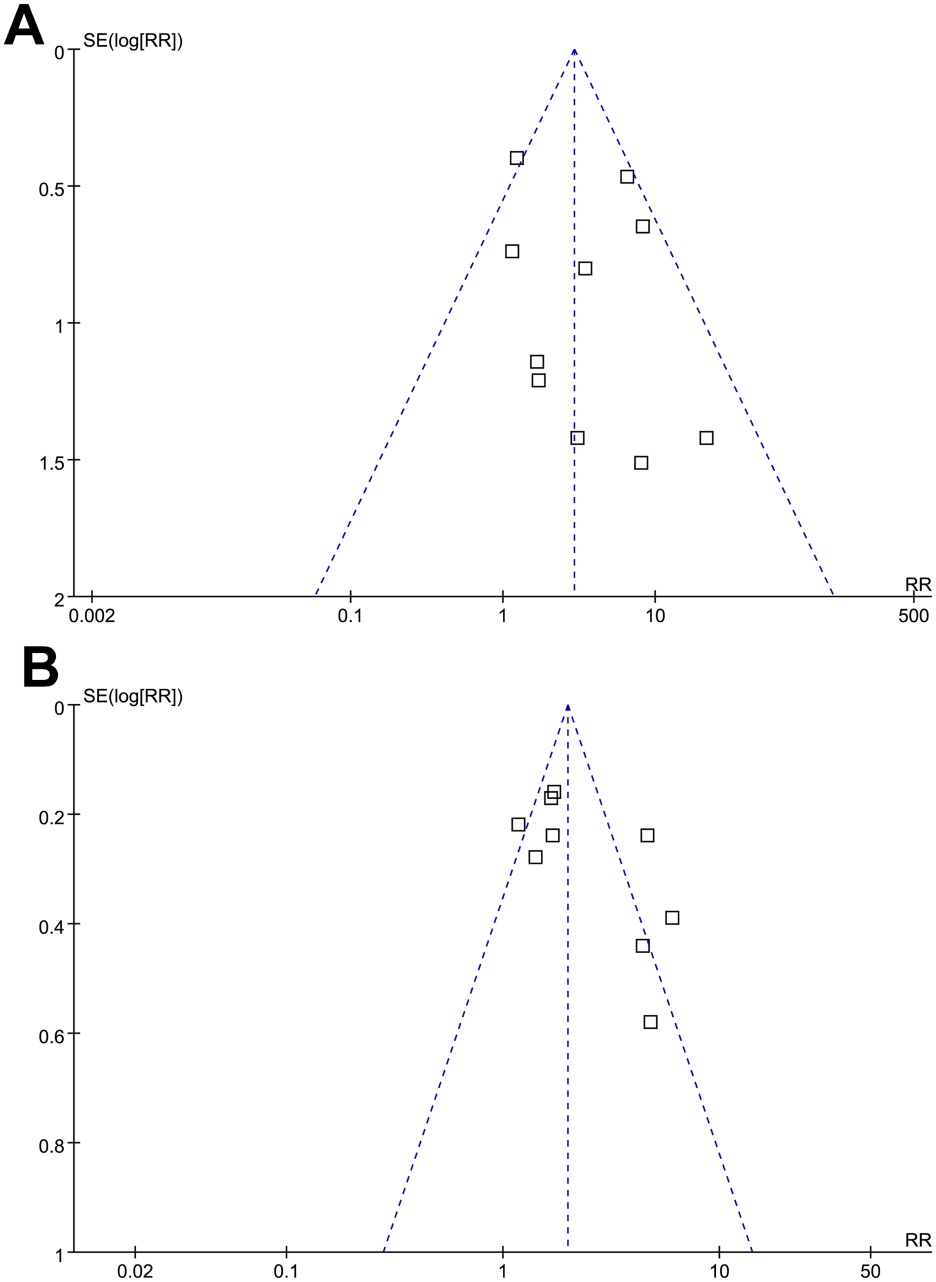
Funnel plots for estimating the potential publication biases underlying the meta-analyses of the associations between the GNRI and short-term and long-term mortality after cardiac surgery. **(A)** Funnel plots for the meta-analysis of the association between the GNRI and short-term mortality after cardiac surgery; **(B)** funnel plots for the meta-analysis of the association between the GNRI and long-term mortality after cardiac surgery. GNRI, Geriatric Nutritional Risk Index.

Figures 7A,B display funnel plots evaluating the association between a low preoperative GNRI and the risk of short- and long-term mortality. These findings were further supported by Egger’s regression analyses, which did not suggest a significant publication bias for the outcomes of short-term (*p* = 0.39) and long-term (*p* = 0.22) mortality. Publication biases for postoperative AKI, wound complications, and overall infection could not be determined due to the limited sample sizes as only four to seven studies were available for these outcomes.

## Discussion

This meta-analysis offers a comprehensive assessment of the relationship between preoperative GNRI and postoperative outcomes in cardiac surgery patients. The findings suggest that a low GNRI is significantly linked to increased short- and long-term mortality, as well as a higher risk of AKI and postoperative infections. However, no significant association was found with wound complications. These results highlight the potential prognostic value of preoperative nutritional status in this patient population.

The association between a low GNRI and increased mortality risk may be explained by several pathophysiological mechanisms. Malnutrition is known to contribute to systemic inflammation, immune dysfunction, delayed wound healing, and a higher susceptibility to infections, all of which can worsen surgical outcomes (38, 39). GNRI is derived from serum albumin levels and body weight, both of which are critical indicators of nutritional and metabolic status. Albumin is a key determinant of oncotic pressure and plays a vital role in transporting essential nutrients and hormones (40). Hypoalbuminemia is associated with chronic inflammation, increased vascular permeability, and impaired recovery from surgical stress (41). Additionally, body weight reflects overall nutritional reserves, and a lower body weight is linked to muscle wasting (42), frailty (43), and reduced physiological reserves (44), which can impair a patient’s ability to recover from the metabolic demands of surgery. Together, these components highlight the role of nutritional status in influencing postoperative survival.

It was noted that one study (25) contributed an extremely wide confidence interval, raising concerns about potential outlier influence in the meta-analysis of the outcome of short-term mortality after cardiac surgery. However, exclusion of this study in a sensitivity analysis did not substantially alter the pooled result for short-term mortality (RR: 2.91, 95% CI: 1.54–4.59, *p* = 0.001; *I*^2^ = 37%), confirming the robustness of the association. Additionally, this study did not appear as an outlier in the funnel plot (Figure 7A), suggesting its influence on the pooled effect size was limited despite the imprecision in its estimate. Besides the sensitivity analyses, subgroup analyses can also offer additional insights into the robustness of the findings. It was found that the association between the GNRI and mortality persisted across different types of cardiac surgeries, including CABG and valvular procedures, suggesting that the prognostic value of the GNRI is not limited to a specific surgical subgroup. While these procedures differ in surgical complexity, duration of cardiopulmonary bypass, and baseline patient characteristics—factors that may influence perioperative metabolic demand and nutritional vulnerability—the consistent association across surgery types highlights the generalizable role of the GNRI as a prognostic marker. This reinforces the relevance of preoperative nutritional assessment regardless of the specific cardiac procedure performed. Moreover, variations in GNRI cutoff values and follow-up durations did not significantly modify the observed associations, reinforcing the consistency of the findings. Differences in study quality, as assessed using the NOS, also did not substantially affect the results, further supporting the reliability of the meta-analysis. However, these results should be interpreted with caution because they were based on study-level data from a limited number of studies, and these results should be validated in large-scale prospective studies.

To further explain the observed heterogeneity, univariate meta-regression analyses were conducted. For long-term mortality, mean age emerged as a significant contributor to heterogeneity. This inverse association suggests that the prognostic impact of a low GNRI may be more pronounced in relatively younger cardiac surgery patients compared to their older counterparts. One possible explanation for this is that in younger patients, malnutrition may reflect a more severe underlying physiological or disease burden, such as cancer cachexia, chronic inflammation, or unrecognized sarcopenia, which would significantly amplify the surgical risks (45). In contrast, older patients often have a chronically low GNRI due to age-related sarcopenia and hypoalbuminemia, which may exert a more modest incremental risk relative to their already elevated baseline mortality (45). Additionally, for short-term mortality, sample size appeared to explain a moderate proportion of the heterogeneity (adjusted *R*^2^ = 23.5%, *p* = 0.095), although this was not statistically significant. This may reflect small-study effects, whereby studies with smaller samples are more susceptible to selection bias, an overestimation of effects, or publication bias. Smaller studies may also be more homogeneous in terms of patient characteristics, institutional practices, or perioperative protocols, leading to more pronounced or variable effect sizes. In contrast, larger studies may average out these differences, leading to more conservative estimates. These findings underscore the complexity of interpreting effect sizes across diverse populations and emphasize the importance of considering both biological and methodological heterogeneity in the synthesis of all available evidence. Taken together, the meta-regression findings suggest that demographic factors (such as age) and study design characteristics (such as sample size) may shape the observed relationship between the GNRI and prognosis of patients after cardiac surgery, further highlighting the need for individualized risk stratification and standardized methodological approaches in future research.

A key finding of this meta-analysis is the differential association of a low GNRI with various postoperative complications. While a significant association was observed with AKI and postoperative infections, the relationship with wound complications was not statistically significant. However, this result should be interpreted cautiously, as it was based on only four studies, and the analysis may have lacked adequate statistical power to detect a true effect. Therefore, the absence of a significant association may reflect insufficient evidence rather than a definitive lack of a relationship. Further studies with larger sample sizes and standardized definitions of wound complications are needed to clarify this potential link. The increased risk of AKI in patients with a low GNRI may be attributable to impaired renal perfusion, systemic inflammation, and oxidative stress, which are common in malnourished individuals (46). Additionally, low albumin levels can compromise endothelial function and lead to microvascular dysfunction, increasing the likelihood of renal injury following cardiac surgery (47). The higher risk of infections in patients with a low GNRI may be linked to impaired immune function, as malnutrition is associated with reduced levels of immunoglobulins and a diminished acute-phase response, making patients more susceptible to infections (48). The lack of a significant association with wound complications may be due to the limited number of studies reporting this outcome, resulting in insufficient statistical power. Future research with larger datasets is needed to clarify this relationship.

The key strengths of this meta-analysis include that it involved a thorough literature search; a large, pooled sample size; and an evaluation of multiple postoperative outcomes, offering a comprehensive assessment of GNRI’s prognostic value in cardiac surgery. The inclusion of both short- and long-term mortality enhances its clinical relevance, while the sensitivity and subgroup analyses reinforce the robustness of the results. Consistency across analytical models and patient subgroups further supports the validity of the conclusions. Despite these advantages, the study has certain limitations that should be noted. Most of the included studies were retrospective in design, making them susceptible to recall bias, selection bias, and residual confounding (49). Therefore, the results of the meta-analysis should be validated in large-scale prospective cohort studies. Furthermore, although most studies adjusted for key clinical variables, the possibility of unmeasured confounders influencing the associations cannot be ruled out. For example, there was variability in the timing of GNRI assessment among the included studies—ranging from at admission to the immediate preoperative period—which may have introduced a confounding effect. Since no standardized timing for GNRI evaluation has yet been established in the cardiac surgical setting, it remains uncertain whether an earlier or later assessment may better reflect perioperative nutritional risk. Despite this variability, the consistent associations observed in our analyses support the robustness of the GNRI, although future studies should clarify the optimal assessment window. Additionally, given that this meta-analysis was based on study-level data rather than individual patient data, we were unable to account for patient-specific factors, such as frailty, preoperative functional status, or the impact of perioperative interventions on outcomes. Another important limitation is the potential for publication bias, as studies with negative results may be less likely to be published. While funnel plots and Egger’s regression test did not indicate significant publication bias for the mortality outcomes, the small number of studies available for some complications limited our ability to formally assess bias in those analyses. Lastly, this meta-analysis was observational in nature and, therefore, could not establish causality between the GNRI and postoperative outcomes.

The findings of this study have important clinical implications. The significant association between the GNRI and adverse postoperative outcomes highlights the potential value of routine preoperative nutritional assessment in patients undergoing cardiac surgery. While the GNRI should not yet be considered a treatment target, its use in risk stratification may help identify high-risk patients who could benefit from enhanced perioperative nutritional support and closer postoperative monitoring. Future research should focus on prospective cohort studies and randomized controlled trials (RCTs) to further evaluate the role of nutritional interventions, particularly those targeting the GNRI, in improving surgical outcomes. Interestingly, studies have shown that preoperative nutritional optimization—including energy–protein supplementation and immunonutrition—can reduce postoperative complications and improve recovery in malnourished surgical patients, including those undergoing cardiac surgery (50, 51). A previous systematic review and meta-analysis of RCTs found that preoperative carbohydrate loading reduced postoperative insulin resistance, ICU stay, and the need for inotropic support in cardiac surgery patients (50). Moreover, expert guidelines also recommend incorporating nutritional support strategies into the care of cardiac surgical patients to improve outcomes (52). While these interventions were not specifically guided by `GNRI, they provide a compelling rationale for exploring whether GNRI-based nutritional interventions can further optimize surgical recovery. Future prospective and interventional studies are needed to evaluate the effectiveness of such strategies. Additionally, studies incorporating individual patient data could provide more granular insights into how specific patient characteristics influence the association between the GNRI and outcomes.

## Conclusion

In conclusion, this meta-analysis demonstrates that a low preoperative GNRI is significantly associated with an increased risk of short-term and long-term mortality, as well as major postoperative complications, such as AKI and infections, in patients undergoing cardiac surgery. Although prospective studies are needed to validate the results, these findings of the meta-analysis support the potential utility of the GNRI as a prognostic tool for surgical risk stratification. Further research is needed to refine the clinical application of the GNRI and explore strategies for optimizing perioperative nutritional management in this patient population.

## Data Availability

The original contributions presented in the study are included in the article/supplementary material, further inquiries can be directed to the corresponding author.
